# Rutin reduces oxidative stress in animals with renovascular hypertension

**DOI:** 10.1186/1753-6561-8-S4-P65

**Published:** 2014-10-01

**Authors:** Rayssa Duarte, Alynne Carvalho, Danilo Gadelha, Valdir Braga

**Affiliations:** 1 Universidade Federal da Paraiba (UFPB), João Pessoa, Brazil

## Introduction

Rutin has been shown be a potent antioxidant that restores impaired vascular reactivity and baroreflex sensitivity in hypertensive rats, mainly by decreasing oxidative stress.

## Objective

This study aimed to evaluate the effect of rutin administration on the baroreflex and oxidative stress in serum of rats with renovascular hypertension (2K1C hypertension model) and their normotensive controls.

## Methods

Twenty-four rats were divided into 4 groups: sham + saline, sham + rutin, 2K1C + saline and 2K1C + rutin. Six weeks after 2K1C surgery, animals presented hypertension compared to the control group (142 ± 2 mmHg versus 121 ± 2 mmHg, n = 8, p <0.05), while there was no change in heart rate. After six weeks, animals were treated with saline or rutin (30 mg/kg/day) by gavage for 14 days. The baroreflex sensitivity was evaluated using intravenous injection of phenylephrine (8 mg/kg) and sodium nitroprusside (25 mg/kg). Lipid peroxidation was measured in serum by thiobarbituric acid reactive species assay (TBRAS).

## Results

Chronic treatment with rutin produced an improvement in baroreflex sensitivity in the 2K1C group, raising it to levels similar to those observed in the Sham + Saline group (-2.55 ± 0.15 vs. -2.70 ± 0.20 bpm.mmHg^-1^, respectively, n = 6, p = 0.05) and higher compared to 2K1C + saline group (-2.55 ± 0.15 vs. -1.74 ± 0.10 bpm.mmHg^-1^, n = 6; p = 0.05). Serum levels of lipid peroxidation in 2K1C rats were higher than in the sham group (5.29 ± 0.93 vs. 4.00 ± 0.01 nmol de MDA/ml respectively, n = 6, p <0.05). Chronic administration of rutin reduced serum levels of lipid peroxidation in hypertensive rats when compared to 2K1C + saline group (2.24 ± 5.29 vs. 0.93 ± 0.55 nmol de MDA/ml, respectively, p < 0.05). No changes were found in the sham group.

## Conclusion

These results suggest that rutin restores baroreflex sensitivity and reduce oxidative stress in rats with renovascular hypertension.

